# Development of the Er-Kay Classification: A Novel Volume-Based Assessment of Cesarean Scar Defects and Their Association with Abnormal Uterine Bleeding

**DOI:** 10.3390/jcm14186592

**Published:** 2025-09-18

**Authors:** Sait Erbey, Fulya Kayikcioglu

**Affiliations:** Department of Gynecology and Obstetrics, Ankara Etlik City Hospital, Varlık Mahallesi, Halil Sezai Erkut Caddesi, Yenimahalle, 06170 Ankara, Turkey; fkayikci@gmail.com

**Keywords:** cesarean scar defect, isthmocele, abnormal uterine bleeding, postmenstrual bleeding, cesarean section

## Abstract

**Objective:** This study aimed to determine the prevalence of isthmocele in patients who had undergone cesarean delivery and to investigate its association with abnormal uterine bleeding (AUB). Additionally, a novel volume-based classification system (Er-Kay Classification) was developed to provide a more precise assessment of cesarean scar defects and their correlation with clinical symptoms. **Material and Methods:** This retrospective, hospital-based cohort study was conducted at Ankara Etlik Zübeyde Hanım Women’s Health Training and Research Hospital between October 2017 and March 2018. A total of 1098 patients who had undergone cesarean delivery and attended follow-up visits were included. Patients were categorized based on the presence of isthmocele (study group: n = 134) and its absence (control group: n = 964). Isthmocele volume was calculated using the formula (Height × Width × Depth)/3, and patients were classified as Grade 1 (≤50 mm^3^) or Grade 2 (>50 mm^3^) based on the novel Er-Kay Classification. Clinical symptoms, including AUB (pre-, inter-, postmenstrual bleeding), dysmenorrhea, dyspareunia, and postcoital bleeding, were compared between groups. Statistical analyses were performed using SPSS 27.0 (NY, USA),with a significance level of *p* < 0.05. **Results:** The prevalence of isthmocele was 12.2% (134/1098). Patients with isthmocele had significantly shorter menstrual cycles compared to those without (26.64 ± 5.35 vs. 28.08 ± 4.97 days, *p* = 0.038). Postmenstrual bleeding (47.0% vs. 4.7%, *p* < 0.001), dysmenorrhea (38.8% vs. 18.3%, *p* < 0.001), and dyspareunia (39.6% vs. 14.7%, *p* < 0.001) were significantly more frequent in the isthmocele group. According to the Er-Kay Classification, intermenstrual bleeding was significantly higher in Grade 2 (23.1%) than in Grade 1 (4.3%) (*p* = 0.001). Similarly, postmenstrual bleeding was more common in Grade 2 (56.9%) than in Grade 1 (37.7%) (*p* = 0.026). No significant differences were found for premenstrual bleeding, dysmenorrhea, or dyspareunia between the Er-Kay Classification groups (*p* > 0.05). **Conclusions:** The findings indicate that isthmocele is significantly associated with AUB, dysmenorrhea, and dyspareunia. The Er-Kay Classification, based on isthmocele volume, provides a more precise assessment of symptom severity, particularly in intermenstrual and postmenstrual bleeding cases. These results suggest that volume-based evaluations should be incorporated into clinical practice for better patient management and diagnosis of cesarean scar defects.

## 1. Introduction

Cesarean section (C-section) is one of the most frequently performed surgical interventions in modern obstetrics, with rates steadily increasing worldwide due to both medical indications and non-medical factors [1,2]. While this procedure has played a critical role in reducing maternal and neonatal morbidity and mortality, its long-term consequences for women’s reproductive health have become an emerging concern [3]. Among these, cesarean scar defects (CSDs), also referred to as isthmocele or uterine niche formation, represent an important clinical entity resulting from abnormal healing of the uterine incision [4,5].

Cesarean scar defects are often asymptomatic but may present with a variety of gynecological and reproductive problems, including abnormal uterine bleeding (AUB), secondary infertility, chronic pelvic pain, and obstetric complications in subsequent pregnancies [6,7]. Postmenstrual spotting, in particular, has been consistently linked with the presence of CSD and is believed to result from impaired menstrual blood clearance due to the anatomical defect [8,9]. Reported prevalence rates of CSD vary widely, ranging from 20% to over 60%, depending on the diagnostic modality used and patient population studied [10,11].

Recent systematic reviews and meta-analyses have confirmed that the presence of CSD significantly increases the risk of AUB, with approximately one in four affected patients experiencing bleeding abnormalities [12]. Moreover, larger and deeper defects are more strongly correlated with postmenstrual and intermenstrual bleeding, suggesting that morphometric characteristics of the defect play a critical role in symptomatology [13,14]. Despite these findings, the majority of existing classification systems rely on two-dimensional parameters such as depth or residual myometrial thickness, which may not fully capture the clinical impact of this inherently three-dimensional defect [15,16].

To address these limitations, there has been growing interest in developing more precise diagnostic and classification strategies, including three-dimensional or volume-based assessments [17,18]. Such approaches may provide a better correlation with clinical outcomes and offer more reliable guidance for patient counseling and management. However, to date, there is no universally accepted classification system that incorporates defect volume as a determinant of symptom severity.

The present study was therefore designed with two main objectives: first, to determine the prevalence of cesarean scar defects (isthmocele) in a large cohort of women with prior cesarean delivery, and second, to investigate the association between these defects and abnormal uterine bleeding. Additionally, we introduce and evaluate a novel volume-based classification system (Er-Kay Classification), hypothesizing that isthmocele volume provides a stronger correlation with clinical symptoms—particularly intermenstrual and postmenstrual bleeding—compared to conventional two-dimensional measures. By establishing this new classification, our study aims to contribute to a more accurate assessment framework for cesarean scar defects and improve clinical decision-making in the management of affected patients.

One of the most significant clinical concerns associated with cesarean scar defects is abnormal uterine bleeding (AUB), particularly postmenstrual spotting [1,2]. The altered uterine anatomy caused by the incomplete healing of the incision site is hypothesized to impair normal menstrual flow, contributing to AUB [3]. Additionally, CSDs may result in secondary infertility, chronic pelvic pain, and complications in future pregnancies, underlining the need for an in-depth investigation into their prevalence and clinical impact [4].

The prevalence of cesarean scar defects varies widely across studies, with reported rates influenced by diagnostic methods, patient characteristics, and the time elapsed since the cesarean section [5]. While some studies emphasize transvaginal ultrasound (TVUS) as an effective tool for detecting these defects, others advocate the use of more advanced imaging modalities, such as saline infusion sonohysterography or magnetic resonance imaging (MRI), to ensure more precise diagnosis and assessment of CSDs [6].

The aim of this study was to determine the prevalence of cesarean scar defects (isthmocele) and their association with abnormal uterine bleeding using ultrasound. In addition, as the first study evaluating isthmocele volume, we aimed to provide novel insights into the literature.

## 2. Materials and Methods

### 2.1. Study Design

This study is a retrospective, hospital-based cohort study conducted at Ankara Etlik Zübeyde Hanım Women’s Health Training and Research Hospital between October 2017 and March 2018. The study was approved by the institutional ethics committee on 26 October 2017, with decision number 90057706-900. Patients who had undergone cesarean delivery within the specified period and attended follow-up visits were evaluated in this study.

### 2.2. Study Population

A total of 2700 patients, identified through the hospital information management system records, were contacted via telephone and invited to participate in the study. Among them, 1114 patients attended the follow-up visit and were included in the study. However, 16 patients were excluded: 10 due to pregnancy at the time of participation, 4 due to the presence of endometrial polyps, and 2 due to the detection of submucosal myomas. Additionally, patients who had undergone uterine surgery other than cesarean section or had medical or organic pathologies that could affect uterine bleeding were not included in the study. The 1098 participants were divided into two groups based on the presence of an isthmocele. The study group consisted of 134 patients diagnosed with an isthmocele, while the control group included 964 patients without isthmocele. Clinical symptoms and abnormal uterine bleeding patterns were compared between the two groups.

### 2.3. Data Collection and Patient Evaluation

The history-taking, clinical examination, and assessments of all patients were conducted by a single physician and recorded in a standardized form. Data collected included patients’ age, gravidity, parity, previous delivery modes, history of abnormal uterine bleeding (pre-, inter-, and postmenstrual bleeding), dysmenorrhea, dyspareunia, postcoital bleeding, fertility desires, and contraceptive methods used. Surgical details such as indications for cesarean section (e.g., fetal distress, arrest of labor, macrosomia) and whether the procedure was performed electively or during labor were retrieved from patient files and the hospital information system. Transvaginal ultrasonography (TVUS) was performed using a GE LOGIQ P5^®^ ultrasound system (GE Healthcare, Chicago, IL, USA) equipped with an E8C^®^ endovaginal 5 MHz probe. During the examination, the cervix, uterus, bilateral adnexal regions, and the Douglas pouch were evaluated. Additionally, the presence of pathologies such as endometrial polyps, submucosal myomas, or irregularities in the endometrial cavity was documented. In patients diagnosed with isthmocele (cesarean scar defect), the dimensions of the defect, including height, width, and depth, were measured and recorded in three planes.

## 3. Er-Kay Classification: A Novel Volume-Based Approach

**Figure 1:**
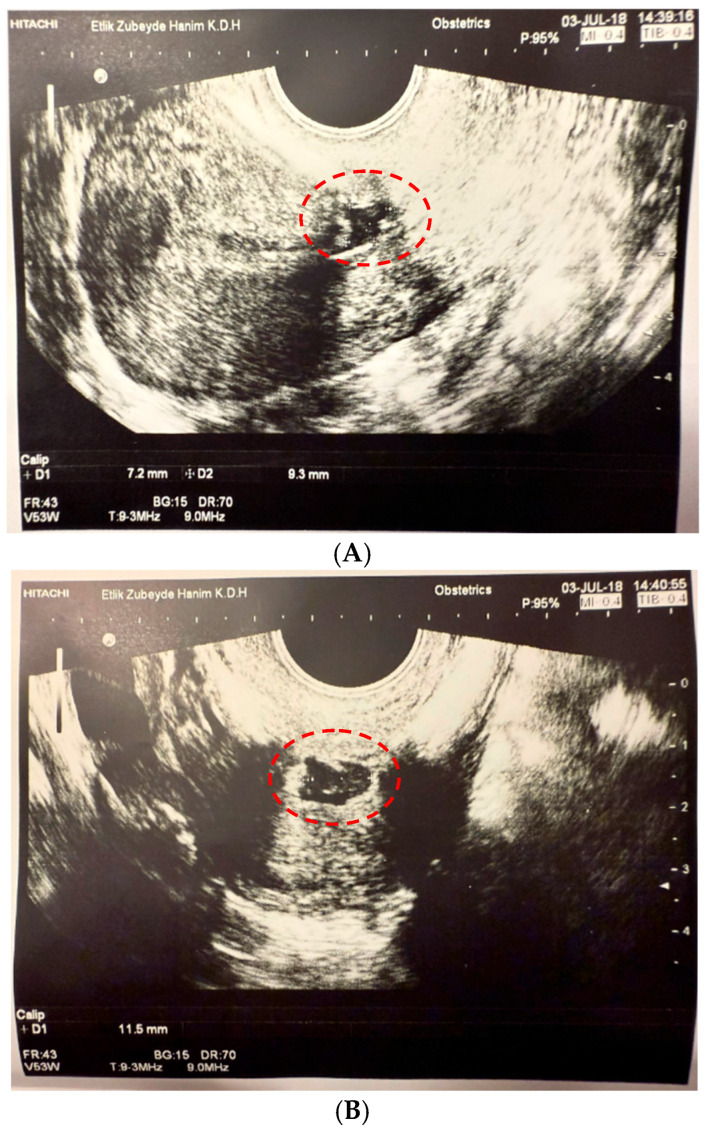
Calculation of Isthmocele Volume in Ultrasonography ((**A**) Height-Depth Measurement (D1*D2), (**B**) Width Measurement (D1)). Height-Depth Measurement is indicated as D1*D2 in panel (**A**), and Width Measurement is indicated as D1 in panel (**B**) (as described in the figure legend). The measured areas are highlighted with red circles on the images.

In our study, a new classification system based on isthmocele volume was developed, differing from existing classifications in the literature, such as Gubbini’s classification [19]. Since the isthmocele generally has an approximately triangular pyramid shape, its volume was calculated using the formula: Volume = (Height × Width × Depth)/3 (Figure 1). Based on the obtained volume values, patients were categorized into two groups. Defects with a volume of 50 mm^3^ or less were classified as Grade 1, while those exceeding 50 mm^3^ were designated as Grade 2. This novel volume-based Er-Kay Classification has facilitated a more comprehensive assessment of cesarean scar defects and enabled a more precise analysis of their correlation with clinical symptoms.

## 4. Study Variables and Endpoints

The primary objective of this study was to determine the prevalence of isthmocele in patients who had undergone cesarean delivery and to investigate its relationship with abnormal uterine bleeding. Secondary objectives included evaluating the impact of cesarean section indications and the number of previous cesarean deliveries on the development of isthmocele. In patients assessed using the Er-Kay Classification, abnormal uterine bleeding patterns such as pre-, inter-, and postmenstrual bleeding, as well as pain-related symptoms like dysmenorrhea and dyspareunia, were analyzed. Additionally, the presence of postcoital bleeding was also evaluated.

## 5. Statistical Analysis

The data analysis was conducted using the SPSS 27.0 (NY, USA) statistical software package. Descriptive statistics were presented as mean ± standard deviation (Mean ± S.D.) for continuous variables and as frequency (n) and percentage (%) for categorical variables. For comparisons of continuous variables between groups, either the independent samples *t*-test or the Mann–Whitney U test was used, depending on the normality assumption. The Chi-square test was applied for categorical variable comparisons, while Fisher’s Exact Test was used when expected frequencies were less than 5. A *p* value < 0.05 was considered statistically significant.

## 6. Results

In our study, isthmocele was detected in 134 out of 1098 patients (12.2%), while 964 patients (87.8%) did not have isthmocele. The cases with isthmocele were divided into two groups based on the volume-based Er-Kay Classification. Isthmocele cases with a volume of 50 mm^3^ or less were classified as Grade 1, comprising 65 patients (48.5%). Cases with a volume greater than 50 mm^3^ were defined as Grade 2 and included 69 patients (51.5%). The mean age of participants was 32.1 ± 5.5 years. On average, participants had 2.5 ± 1.16 pregnancies (gravida) and 1.87 ± 0.82 live births (parity). The mean menstrual cycle frequency was 27.91 ± 5.04 days, and the average menstrual duration was 5.69 ± 1.94 days. Participants used an average of 3.42 ± 1.53 pads per day during their menstruation.

The descriptive characteristics of the evaluated patients are presented in Table 1.

The frequency of menstruation was significantly different between patients with and without isthmocele (26.64 ± 5.35 vs. 28.08 ± 4.97 days, *p* = 0.038). This indicates that those with isthmocele had slightly shorter menstrual cycles. Other parameters, such as the number of cesareans, menstrual duration, and the number of pads used per day, did not show statistically significant differences (*p* > 0.05) (Table 2).

The key findings indicate that intermenstrual bleeding was significantly more frequent in patients with isthmocele (13.4%) compared to those without (8.2%) (*p* = 0.045). Postmenstrual bleeding showed a highly significant difference, occurring in 47.0% of patients with isthmocele versus 4.7% of those without (*p* < 0.001). Additionally, dysmenorrhea was reported by 38.8% of patients with isthmocele and 18.3% of those without (*p* < 0.001). Dyspareunia was also notably more common among patients with isthmocele (39.6%) than those without (14.7%) (*p* < 0.001) (Table 3).

The results demonstrate a significant relationship between the time of cesarean section and the presence of isthmocele. Patients who had a cesarean during labor exhibited a higher incidence of isthmocele (21.1%) compared to those who underwent elective cesarean sections (6.5%), with a *p* value of <0.001. In contrast, other factors showed no statistically significant association with the development of isthmocele (*p* > 0.05) (Table 4).

The measurements of cesarean scar defects revealed that the mean volume was 51.9 ± 18.7 mm^3^, with the interquartile range between 38.5 mm^3^ and 61.9 mm^3^. The area of the defect averaged 14.34 ± 4.1 mm^2^. The height, width, and depth of the scar defect were also assessed, with mean values of 5.2 ± 0.6 mm, 5.5 ± 1.1 mm, and 5.36 ± 1.0 mm, respectively (Table 5).

The comparison of menstrual cycle characteristics between Grade 1 and Grade 2 groups according to the Er-Kay classification showed no statistically significant differences (Table 6).

Comparing the symptoms between the Grade 1 and Grade 2 groups based on the Er-Kay classification, significant differences were revealed in some parameters. Intermenstrual bleeding was more frequent in the Grade 2 group (23.1%) compared to the Grade 1 group (4.3%), with a *p* value of 0.001. Postmenstrual bleeding was also significantly higher in the Grade 2 group (56.9%) than in the Grade 1 group (37.7%), with a *p* value of 0.026. However, other symptoms such as premenstrual bleeding, dysmenorrhea, and dyspareunia did not show statistically significant differences between the two groups (*p* > 0.05) (Table 7).

The table shows symptom distribution across three grades based on the Gubbini classification. Intermenstrual bleeding presents a statistically significant difference (*p* = 0.017), occurring in 10.3% of Grade 1, 11.4% of Grade 2, and increasing sharply to 50.0% in Grade 3 patients. Although postmenstrual bleeding is more frequent in higher grades—47.1% in Grade 1, 40.0% in Grade 2, and 100.0% in Grade 3—the *p* value of 0.054 indicates it is not statistically significant. Similarly, dysmenorrhea shows a trend, with 39.1% in Grade 1, 34.3% in Grade 2, and 83.3% in Grade 3, but this difference is also not statistically significant (*p* = 0.074). No significant differences were found for premenstrual bleeding (*p* = 0.093) or dyspareunia (*p* = 0.889) across the grades (Table 8).

A statistically significant difference was observed in diaper use concerning isthmocele width (*p* = 0.008). In patients with intermenstrual bleeding, the mean isthmocele width was 6.0 ± 1.1 mm, which was significantly higher compared to those without intermenstrual bleeding (*p* = 0.045). Similarly, in patients with premenstrual bleeding, isthmocele depth was found to be significantly higher (7.0 ± 2.1 mm, *p* = 0.046). Postmenstrual bleeding was significantly associated with both isthmocele height (*p* = 0.012) and depth (*p* = 0.015). Dysmenorrhea showed a significant difference concerning isthmocele height (*p* = 0.009), with patients experiencing dysmenorrhea having a greater isthmocele height. Conversely, no statistically significant differences were found between isthmocele dimensions and dyspareunia (*p* = 0.242) or postcoital bleeding (*p* = 0.577) (Table 9).

## 7. Discussion

In recent years, increasing attention has been given to the clinical outcomes of cesarean scar defects (isthmocele) and their impact on women’s health [9]. Studies aimed at understanding the prevalence of isthmocele and its relationship with symptoms provide significant contributions to the existing literature [1]. In this study, the presence of isthmocele in patients with a history of cesarean delivery was found to be associated with abnormal uterine bleeding and pain symptoms. Patients diagnosed with isthmocele exhibited a marked increase in the frequency of intermenstrual and postmenstrual bleeding. Additionally, pain-related symptoms such as dysmenorrhea and dyspareunia were more prevalent in the isthmocele group. It was also determined that cesarean sections performed during labor had a significant effect on the development of isthmocele. With the novel volume-based Er-Kay Classification developed in this study, we were able to categorize isthmocele cases based on their volume and analyze their relationship with symptoms in greater detail. According to this first-ever volume-based classification in the literature, larger-volume isthmoceles (Grade 2) were associated with a higher incidence of intermenstrual and postmenstrual bleeding. Our findings suggest that this new classification system is both appropriate and beneficial in the clinical assessment of isthmocele and in determining its association with symptoms.

The Er-Kay Classification demonstrates a stronger performance in predicting intermenstrual and postmenstrual bleeding symptoms. By employing a volume-based approach, it better reflects the actual size of the scar defect and establishes a stronger correlation with clinical symptoms. In contrast, the Gubbini Classification, although useful for symptom classification, relies on two-dimensional measurements, which may not always accurately predict the severity and frequency of symptoms. Particularly, it has limitations in identifying cases where intermenstrual and postmenstrual bleeding is more severe. In conclusion, the Er-Kay Classification provides more meaningful results in symptom prediction and serves as a more useful clinical guide by considering the volume of the scar defect. These findings suggest that the Er-Kay Classification is a more effective and reliable tool for clinical assessments.

Antila et al. found no significant difference in the occurrence of a niche, defined as an anechoic defect of at least 2.0 mm depth at the cesarean section scar site detected via SHG, between the two study groups (7.2% vs. 7.2%, *p* = 0.998) [8]. The duration of extended menstrual bleeding in women with a niche has been reported to range from 11.2 to 16.1 days [10,11,12,13]. The prevalence of spotting after CS in women with a niche varies from 20.0% to 60.8% in general populations, while it is notably lower (8.3%) in those without a niche [14,16]. Most studies confirm an association between the presence of a niche and spotting, with the exception of a study by Gozzi et al., which involved 546 participants and found no statistically significant difference between those with and without a niche using TVUS (11.6% vs. 9.8%, *p* = 0.852) [15]. Furthermore, several studies indicate that as the volume of the niche increases, the likelihood of experiencing symptoms such as spotting, abnormal uterine bleeding (AUB), and prolonged menstruation also rises [17,18]. The ratio of niche depth to the thickness of the adjacent uterine wall, as measured by TVS, is also considered an important parameter in predicting the development of these symptoms.

The impact of cesarean scar defect (CSD) on abnormal uterine bleeding (AUB) has been a subject of increasing research interest in recent years [20,21]. However, the use of different methodological approaches and definitions in various studies examining this relationship complicates the comparison of results. In a comprehensive systematic review by Murji et al., existing research findings on the association between CSD and AUB were synthesized. The review highlighted significant discrepancies in the definition and measurement of CSD, as well as variations in the terminology used for AUB. It was reported that approximately one-quarter of patients with CSD experience AUB, and the presence of CSD triples the risk of developing AUB. Notably, CSD has been associated with early intermenstrual bleeding, and larger defects were found to be linked to more severe bleeding symptoms [3]. In a 2011 study by Gubbini et al., the prevalence of postmenstrual abnormal uterine bleeding in patients with isthmocele was reported to be 41% [22]. Similarly, Wang et al. found the prevalence of postmenstrual bleeding in isthmocele cases to be 63.8% [16]. In our study, the prevalence of postmenstrual bleeding in patients diagnosed with isthmocele was found to be 47%, which was significantly higher compared to the 4.7% prevalence in patients without isthmocele. Additionally, according to the Er-Kay Classification, the frequency of postmenstrual bleeding was 37.7% in the Grade 1 isthmocele group, whereas it increased to 56.9% in the Grade 2 group, with this difference being statistically significant. These findings indicate that as the volume of the isthmocele increases, the frequency of postmenstrual bleeding also rises. Our results are consistent with previous studies in the literature, reinforcing the impact of isthmocele on abnormal uterine bleeding and emphasizing the clinical significance of the Er-Kay Classification in patient evaluation.

Isthmocele is inherently a three-dimensional defect; therefore, area measurements based solely on two-dimensional parameters may not fully reflect its actual size and clinical impact [23]. Existing classifications in the literature primarily rely on two-dimensional measurements, such as width and depth, which fail to account for the volumetric effect of the defect. To address this limitation, we measured the three-dimensional parameters of isthmocele, calculated its volume, and classified patients accordingly. In our novel volume-based Er-Kay Classification, defects ≤50 mm^3^ were categorized as Grade I, while those >50 mm^3^ were classified as Grade II. This approach provides a more accurate representation of the actual size of the defect and its correlation with clinical symptoms. In our study, patients with Grade II isthmocele exhibited a significantly higher prevalence of postmenstrual and intermenstrual bleeding. This finding suggests that an increase in defect volume is associated with greater severity and frequency of symptoms. On the other hand, no significant difference was observed between the two groups regarding dysmenorrhea and dyspareunia, indicating that other factors may also contribute to the development of these symptoms. In conclusion, to better understand the mechanism by which isthmocele leads to abnormal uterine bleeding and to improve patient evaluation, we propose that a volume-based classification is more appropriate than traditional area-based methods. This volume-based approach allows clinicians to better assess the clinical significance of isthmocele and make more informed decisions in patient management.

In a 2021 study, Casadio et al. calculated the volume of cesarean scar defect (CSD) in symptomatic patients using the prolate ellipsoid formula [24]. Their findings indicated that the mean CSD volume was 503.9 ± 183.6 mm^3^ in patients with abnormal uterine bleeding (AUB), compared to 364.4 ± 83.1 mm^3^ in those without AUB. Among patients experiencing pelvic pain, the mean CSD volume was 661.1 ± 124.4 mm^3^, whereas it was 388.7 ± 122.3 mm^3^ in those without pain. Similarly, in patients with dyspareunia, the mean CSD volume was 515.5 ± 188.4 mm^3^, while it was 449.8 ± 166.3 mm^3^ in those without this complaint. For dysmenorrhea, the mean CSD volume was 535.5 ± 200.6 mm^3^ in affected patients, compared to 446.2 ± 151.3 mm^3^ in those without dysmenorrhea. While Casadio et al. compared CSD volume based on symptom presence, their study did not establish a classification system based on a specific volume threshold. In contrast, recognizing that isthmocele is a three-dimensional defect, we developed a volume-based classification system. In our proposed Er-Kay Classification, CSDs were categorized as Grade 1 (≤50 mm^3^) and Grade 2 (>50 mm^3^). This classification aimed to more precisely define the relationship between CSD volume and clinical symptoms. Our findings revealed that the prevalence of intermenstrual bleeding was 23.1% in the Grade 2 group, compared to 4.3% in the Grade 1 group. Similarly, postmenstrual bleeding was observed in 56.9% of Grade 2 cases, whereas it was 37.7% in Grade 1 cases. These results indicate that as CSD volume increases, the frequency of abnormal uterine bleeding symptoms also rises. By introducing this volume-based cut-off classification, our study provides a novel perspective on the clinical significance of CSD volume in patient evaluation and management.

Our findings reinforce that cesarean scar defects (isthmocele) are strongly associated with abnormal uterine bleeding (AUB) and pain symptoms, and they highlight the clinical value of a volume-based assessment. In our cohort, larger-volume defects—captured by the Er-Kay Classification—were linked to higher rates of intermenstrual and postmenstrual bleeding, aligning with contemporary evidence that greater defect burden predicts more pronounced bleeding phenotypes [3,24,25]. Beyond two-dimensional measurements, incorporating defect volume may therefore improve risk stratification and patient counseling.

From a diagnostic standpoint, multimodal imaging strategies are increasingly leveraged to characterize defect morphology and to anticipate treatment response; recent work suggests that preoperative MRI parameters may help predict clinical cure in AUB attributed to prior cesarean scar defects [26]. With respect to management, both hysteroscopic and laparoscopic isthmoplasty appear effective for controlling AUB, while emerging comparative data indicate laparoscopy may confer advantages in infertility contexts [27]. Nevertheless, recent commentaries emphasize that high-quality randomized data linking surgical correction to long-term prognosis remain limited, underscoring the need for careful, individualized decision-making [28].

Looking ahead, standardization remains a critical gap. Heterogeneity in how AUB and cesarean scar defects are defined continues to impede synthesis of the literature; calls for consensus definitions and harmonized reporting have been reiterated in 2024 [25]. We propose that future multicenter prospective studies should validate volumetric thresholds (e.g., the Er-Kay cut-offs) against patient-reported outcomes and reproductive endpoints, compare surgical approaches head-to-head in randomized designs, and evaluate imaging-based predictors of treatment response. Until such data mature, integrating a pragmatic volume-based classification alongside symptom profiles and reproductive goals may offer clinicians a more nuanced framework for counseling and management.

This study has certain limitations that should be acknowledged. First, the retrospective design introduces the potential for selection bias, as participation depended on patients attending follow-up visits. Second, because data were derived from a single tertiary hospital, the findings may not be fully generalizable to broader populations with different demographic or obstetric characteristics. Third, although we assessed short-term clinical outcomes, the lack of long-term follow-up prevents us from evaluating the progression of cesarean scar defects, their impact on fertility outcomes, or the durability of symptom patterns over time. Finally, while ultrasonographic measurements were standardized and performed by a single operator to minimize interobserver variability, the absence of cross-validation with other imaging modalities (e.g., MRI, saline infusion sonohysterography) may have limited the robustness of the defect characterization. Future multicenter, prospective studies with long-term follow-up and multimodal imaging are warranted to confirm and expand upon our findings.

## 8. Conclusions

In conclusion, our study demonstrates a significant association between cesarean scar defects and both abnormal uterine bleeding and pain-related symptoms, underscoring the clinical relevance of these defects. By introducing the Er-Kay Classification, the first volume-based system in the literature, we provide a novel framework that more accurately reflects symptom burden compared to purely morphological assessments. Larger-volume defects were shown to correlate with increased rates of intermenstrual and postmenstrual bleeding, highlighting the importance of volumetric evaluation for improved risk stratification. Although limited by its retrospective, single-center design and the absence of long-term follow-up data, this study emphasizes the potential value of integrating volume-based assessment into clinical practice. Such an approach may enhance diagnostic precision, support individualized patient counseling, and guide the selection of optimal management strategies.

## Figures and Tables

**Table 1:** Descriptive characteristics of the patients.

	Minimum	Maximum	Mean ± S.D. *	Median
Age	21	44	32.05 ± 5.48	32
Gravida	1	8	2.5 ± 1.16	2
Parity	1	4	1.87 ± 0.82	2
Abortion	0	3	0.53 ± 0.73	0
Vaginal delivery	0	3	0.35 ± 0.63	0
Cesarean section	1	3	1.52 ± 0.64	1
Menstrual cycle length (days)	15	60	27.91 ± 5.04	28
Menstrual duration (days)	2	15	5.69 ± 1.94	5
Daily pad usage	1	10	3.42 ± 1.53	3

**Abbreviations:** * S.D.: standard deviation.

**Table 2:** Comparison of clinical characteristics between patients with and without isthmocele.

	Isthmocele (n = 134)	No Isthmocele (n = 964)	
	Mean ± S.D.	Mean ± S.D.	
Previous Cesarean Section	1.56 ± 0.64	1.52 ± 0.64	0.459
Frequency of menstruation (days)	26.64 ± 5.35	28.08 ± 4.97	**0.038**
Menstrual duration (days)	5.78 ± 1.89	5.68 ± 1.95	0.224
Number of pads per day	3.46 ± 1.7	3.41 ± 1.51	0.695

The data are presented as mean ± standard deviation. Statistically significant *p* values are indicated in bold.

**Table 3:** Assessment of Symptoms Based on the Presence of Isthmocele.

	Isthmocele (n = 134)	No Isthmocele (n = 964)	
	Count (%)	Count (%)	
Excessive menstrual bleeding **	4 (3.0)	13 (1.3)	0.143 ^a^
Intermenstrual Bleeding	18 (13.4)	79 (8.2)	**0.045 ^b^**
Premenstrual Bleeding	4 (3.0)	29 (3.0)	1.000 ^a^
Postmenstrual Bleeding	63 (47.0)	45 (4.7)	**<0.001 ^b^**
Postcoital Bleeding	3 (2.2)	37 (3.8)	0.465 ^a^
Dysmenorrhea	52 (38.8)	176 (18.3)	**<0.001 ^b^**
Dyspareunia	53 (39.6)	142 (14.7)	**<0.001 ^b^**

The data are presented as count and percentage. Statistically significant *p* values are indicated in bold. ** **Excessive menstrual bleeding** refers to severe cases where the use of diapers, night-time pads, or protective bedding was required, or where the bleeding resulted in anemia. ^a^: Fisher’s Exact Test, ^b^: Chi-square test.

**Table 4:** Evaluation of Isthmocele Based on Cesarean Section Characteristic and Type.

		Isthmocele (n = 134)	No Isthmocele (n = 964)	
		Count (%)	Count (%)	
Indication for C/S	Fetal Distress	32 (13.3%)	209 (86.7%)	0.072
	NP Labor	4 (6.6%)	57 (93.4%)	
	Macrosomia	0 (0%)	36 (100%)	
	Breech Pre	4 (5.8%)	65 (94.2%)	
	CPD	14 (14.3%)	84 (85.7%)	
	Previous C/S	62 (13%)	415 (87%)	
	Others	18 (15.5%)	98 (84.5%)	
C/S Timing	C/S During Labor	91 (21.1%)	341 (78.9%)	**<0.001**
	Elective C/S	43 (6.5%)	623 (93.5%)	
Number of C/S	1	538 (88.5%)	70 (11.5%)	0.736
	2	351 (86.9%)	53 (13.1%)	
	3	75 (87.2%)	11 (12.8%)	

The data are presented as count and percentage. Statistically significant *p* values are indicated in bold. Abbreviations: C/S: Cesarean Section, CPD: Cephalopelvic Disproportion, NP Labor: Non-progressive Labor.

**Table 5:** Presentation of Different Measurements of Cesarean Scar Defect.

	Quartile 25	Quartile 75	Mean ± S.D.
Height (mm)	4.8	5.6	5.2 ± 0.6
Width (mm)	4.7	6.3	5.5 ± 1.1
Depth (mm)	4.8	5.9	5.36 ± 1.0
Area (mm^2^)	12.0	17.5	14.34 ± 4.1
Volume (mm^3^)	38.5	61.9	51.9 ± 18.7

The data are presented as quartile 25, quartile 75, and mean ± standard deviation (S.D.). Abbreviations: S.D.: Standard Deviation.

**Table 6:** Evaluation of Menstrual Cycle Characteristics Based on the Er-Kay Classification.

	Er-Kay Grade 1 (n = 65)	Er-Kay Grade 2 (n = 69)	
	Mean ± S.D.	Mean ± S.D.	*p* Value
Frequency of menstruation (days)	26.7 ± 5.7	26.6 ± 5.0	0.873
Menstrual duration (days)	5.7 ± 2.2	5.8 ± 1.5	0.417
Number of pads per day	3.2 ± 1.7	3.7 ± 1.7	0.105

The data are presented as mean ± standard deviation (S.D.). Abbreviations: S.D.: Standard Deviation.

**Table 7:** Comparison of Symptoms Based on the Er-Kay Classification.

	Grade 1 (n = 65)	Grade 2 (n = 69)	
	Count (%)	Count (%)	
Intermenstrual Bleeding	3 (4.3)	15 (23.1)	**0.001**
Premenstrual Bleeding	1 (1.4)	3 (4.6)	0.282
Postmenstrual Bleeding	26 (37.7)	37 (56.9)	**0.026**
Dysmenorrhea	28 (40.6)	24 (36.9)	0.664
Dyspareunia	30 (43.5)	23 (35.4)	0.338

The data are presented as count and percentage. Statistically significant *p* values are indicated in bold.

**Table 8:** Comparison of Symptoms Based on the Gubbini Classification.

	Grade 1 (n = 87)	Grade 2 (n = 41)	Grade 3 (n = 6)	
	Count (%)	Count (%)	Count (%)	*p* Value
Intermenstrual Bleeding	9 (10.3)	4 (11.4)	3 (50.0)	**0.017**
Premenstrual Bleeding	1 (1.1)	3 (8.6)	0 (0.0)	0.093
Postmenstrual Bleeding	41 (47.1)	14 (40.0)	6 (100.0)	0.054
Dysmenorrhea	34 (39.1)	12 (34.3)	5 (83.3)	0.074
Dyspareunia	37 (42.5)	14 (40.0)	2 (33.3)	0.889

The data are presented as count and percentage. Statistically significant *p* values are indicated in bold.

**Table 9:** Association Between Isthmocele Dimensions and Symptoms.

		Isthmocele Height	Isthmocele Width	Isthmocele Depth
		Mean ± S.D.	Mean ± S.D.	Mean ± S.D.
Need for Diapers	No	5.2 ± 0.6	5.5 ± 1.1	5.4 ± 1.0
	Yes	4.9 ± 0.4	4.3 ± 0.3	5.4 ± 0.5
*p* value		0.189	**0.008**	0.808
Intermenstrual Bleeding	No	5.1 ± 0.7	5.4 ± 1.1	5.3 ± 1.0
	Yes	5.4 ± 0.4	6.0 ± 1.1	5.5 ± 0.7
*p* value		0.146	**0.045**	0.090
Premenstrual Bleeding	No	5.2 ± 0.6	5.4 ± 1.1	5.3 ± 0.9
	Yes	5.2 ± 0.4	6.0 ± 1.2	7.0 ± 2.1
*p* value		0.880	0.339	**0.046**
Postmenstrual Bleeding	No	5.1 ± 0.6	5.4 ± 1.0	5.2 ± 0.9
	Yes	5.3 ± 0.6	5.6 ± 1.3	5.6 ± 1.0
*p* value		**0.012**	0.770	**0.015**
Dysmenorrhea	No	5.1 ± 0.6	5.5 ± 1.0	5.3 ± 1.0
	Yes	5.4 ± 0.7	5.5 ± 1.2	5.4 ± 1.0
*p* value		**0.009**	0.824	0.623
Dyspareunia	No	5.1 ± 0.7	5.6 ± 1.1	5.3 ± 1.0
	Yes	5.2 ± 0.6	5.3 ± 1.2	5.4 ± 1.1
*p* value		0.514	0.242	0.949
Postcoital Bleeding	No	5.2 ± 0.6	5.5 ± 1.1	5.4 ± 1.0
	Yes	5.2 ± 0.1	5.7 ± 0.2	5.2 ± 0.8
*p* value		0.755	0.577	0.547

## Data Availability

The data supporting the findings of this study are available from the corresponding author, S.E., upon reasonable request. Data access is subject to the approval of the institutional ethics committee and patient confidentiality agreements.
